# Lifemap: Exploring the Entire Tree of Life

**DOI:** 10.1371/journal.pbio.2001624

**Published:** 2016-12-22

**Authors:** Damien M. de Vienne

**Affiliations:** Univ Lyon, Université Claude Bernard Lyon 1, CNRS, Laboratoire de Biométrie et Biologie Evolutive, UMR5558, Villeurbanne, Lyon, France

## Abstract

The Tree of Life (ToL) is meant to be a unique representation of the evolutionary relationships between all species on earth. Huge efforts are made to assemble such a large tree, helped by the decrease of sequencing costs and improved methods to reconstruct and combine phylogenies, but no tool exists today to explore the ToL in its entirety in a satisfying manner. By combining methods used in modern cartography, such as OpenStreetMap, with a new way of representing tree-like structures, I created Lifemap, a tool allowing the exploration of a complete representation of the ToL (between 800,000 and 2.2 million species depending on the data source) in a zoomable interface. A server version of Lifemap also allows users to visualize their own trees. This should help researchers in ecology and evolutionary biology in their everyday work, but may also permit the diffusion to a broader audience of our current knowledge of the evolutionary relationships linking all organisms.

## Introduction

An exhaustive knowledge of the evolutionary relationships linking all organisms (the whole biodiversity) would produce a tree-like structure, referred to as the Tree of Life (ToL). The decrease of DNA sequencing costs [1] associated with improved phylogenetic and phylogenomic methods for reconstructing phylogenetic trees [2–4] helped resolve, in the recent years, many parts of this ToL. The aggregation of these portions produces a phylogenetic classification scheme describing the evolutionary relationships among the species under consideration [5]. The NCBI taxonomy is one of these classifications, describing relationships among ~1.4 million species (as of Oct 2016), that is widely used by biologists. Another version of the ToL, the Open Tree of Life (OTOL), was published recently [6]. It adds to the taxonomy some evolutionary information retrieved from published phylogenetic trees, in order to provide a more accurate and more comprehensive description of the ToL. It currently contains around 2.2 million species.

Despite huge ongoing efforts to assemble the ToL [6,7], no tool exists today to explore it entirely and interactively in a satisfying manner. More generally, the visualization of very large trees still remains a challenge [8]. A parallel has been drawn earlier between what has been achieved in cartography with the development of Google maps or OpenStreetMaps [9] (OSM), and what could be done for exploring the ToL [8,10]. The logic behind the use of the cartographic paradigm for visualizing a taxonomy is straightforward: like geographic entities (countries, regions, cities, etc.), taxonomic levels have names (kingdoms, families, classes, orders, genera, etc.) and are nested within each other. Consequently, it is possible to propose a visualization where zooming in increases the level of detail displayed while hiding upper-level information. This idea was first exploited in a tool called OneZoom [10] that allows visualizing, on demand, large trees by zooming and panning. This tool introduced the concept of deep zooming for visualizing large phylogenies (along with [11]), which motivated other research along the same line, including the present one. The main limitation of OneZoom that makes it inappropriate for visualizing the complete ToL is that the fractal representation it uses prevents the presence of multifurcations in the trees (one node connected to more than two descendants), which is very common (83% of the nodes in the NCBI taxonomy and 72% in the OTOL are not binary).

## Three Versions of the Tree of Life

I developed Lifemap, a tool largely inspired by the technology developed for cartography that is free of the limitation described above. Its approach differs from that of OneZoom [10], both in the representation of the tree and in the way images are displayed and interacted with on the screen. This allows a fast and smooth exploration of the biggest tree ever proposed on a single page for exploration.

**Fig 1:**
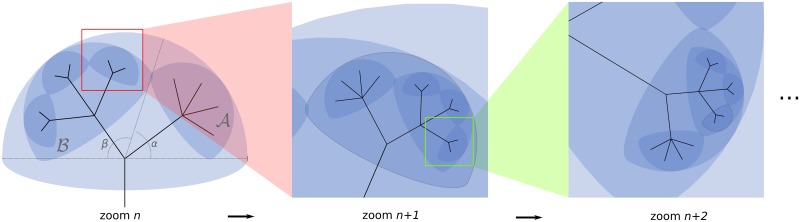
Three successive zoom levels in Lifemap illustrating the half-circle representation employed. Each clade is represented by a half-circle whose size depends on the relative number of species in the clade as compared to its sister clades at a given level. Note that these proportions are not respected at the tree root where the three superkingdoms are arbitrarily given the same size. Computation of the size of each half-circle is based on the angle they are associated with (α and β on the first panel): if *n*_*A*_ and *n*_*B*_ are the number of species in clades A and B, respectively, the angles in degrees are computed as follows: $\alpha =180\times \sqrt{n_{A}}/\left(\sqrt{n_{A}}+\sqrt{n_{B}}\right)$ and $\beta =180\times \sqrt{n_{B}}/\left(\sqrt{n_{A}}+\sqrt{n_{B}}\right)$. The square root reduces the difference in half-circle sizes between very small and very large groups. At every level, the half-circles (clades) are randomly distributed within their parental half-circle.

Lifemap uses a representation inspired by Treemaps, a method developed in the field of computer sciences in the early 90s for turning file system tree structures (directories in a computer) into a planar space-filling map [12] for clearer visualization. In Treemaps, directories are represented by rectangles that are recursively split in as many subrectangles as there are subdirectories, leading to a fully-filled map. The size of the rectangles is proportional to the number of files in each directory. This approach cannot be directly used to visualize the ToL because it is incompatible with the representations of links between the nodes, which is important in an evolutionary context where branches represent time and must be visible. I, however, kept the idea of filling the map recursively, using a base shape whose size is proportional (but with a square root transformation) to the number of elements in it. In Lifemap, this base shape is a half-circle, and the way these half-circles are arranged within and between each other (Fig 1) ensures that there is space to draw all the branches and guarantees that the branches never intersect (unlike other solutions, [13]).

**Fig 2:**
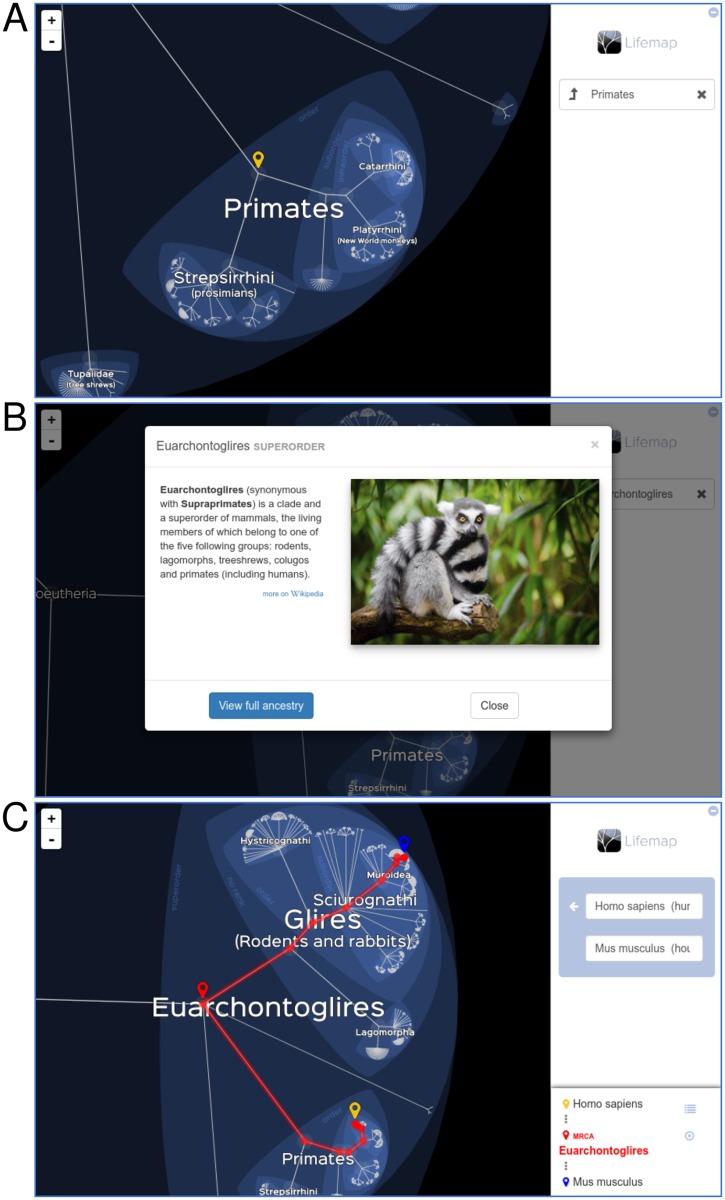
Screenshots of the Lifemap tool. (**A**) Example of the appearance of Lifemap when zooming to the primates order. (**B**) Example of information displayed in the general public version when clicking on a node. (**C**) Visualization of the path between two taxa. Lemur image credit: Mathias Appel, Flickr (https://flic.kr/p/FKtBbU).

Lifemap comes in three versions that differ by the tree that is displayed and the information that is associated with tips and nodes when clicking. The general public version (Fig 2A) displays a reduced NCBI taxonomy obtained by removing nonidentified clades and all taxa below the species level. When clicking on nodes or tips, a short description and a picture are displayed (Fig 2B). Pictures and text are obtained from Wikipedia. If no Wikipedia page exists or if a picture is lacking, the user can click on a link to contribute to Wikipedia for these specific taxa by creating a page, modifying the text, and/or adding a picture. Lifemap should thus help identify missing pages and improve the quality and quantity of pages dedicated to clades and species in Wikipedia.

The NCBI version, named “Lifemap NCBI,” displays the whole NCBI taxonomy and is updated every week. When clicking on a node, the user can (i) get additional information about the current taxa (taxid, number of species), (ii) reach the NCBI web page corresponding to the node, and (iii) download the corresponding subtree in parenthetic format for further analysis. In this version, the user can also add a layer to the tree to visualize at each node the number of fully sequenced genomes.

The third version is named “Lifemap OTOL.” It displays the latest OTOL synthetic tree and will be updated every time a new version is released. The information displayed when clicking on the nodes is similar to the one available in the two others: Wikipedia picture and description, taxonomy code, possibility to download the subtree in parenthetic format, and taxonomic sources of information for each node. Other information that can be displayed as layers on Lifemap will be added in the future in response to user's suggestions and requests in the different versions.

Finally, all three versions give the possibility to compute, visualize, and explore “paths” in the ToL (Fig 2C). This is done either by choosing a source and a destination taxa or by clicking the “view full ancestry” button associated with each node. In the latter case, the destination is set as the root of the tree. The path is computed instantly and highlighted on the tree. The most recent common ancestor (MRCA) is indicated with a marker, and the list of taxa encountered in the route from the source to the destination is returned.

## A Virtual Machine for Exploring Any Tree

**Fig 3:**
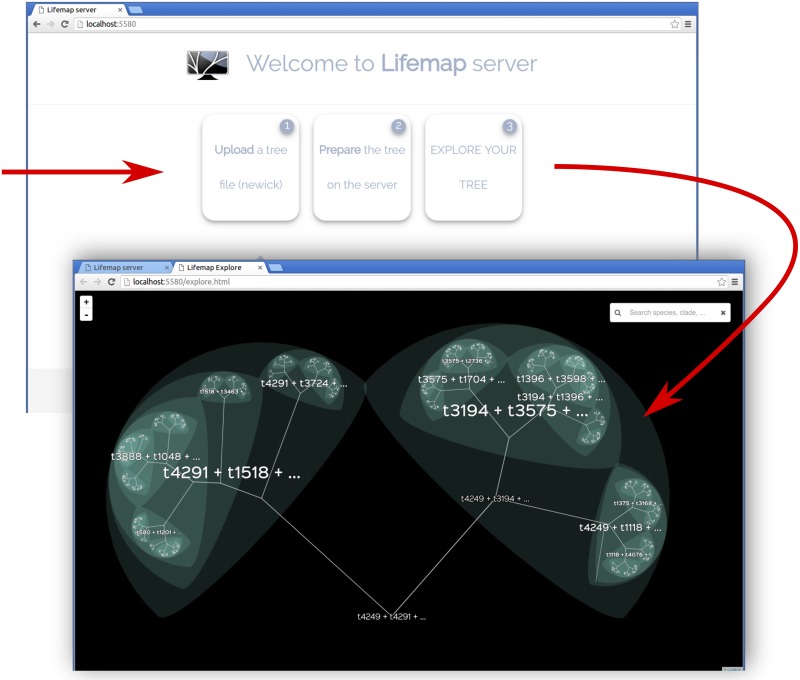
Screenshots of the Lifemap server. Visualizing a tree with the Lifemap server is a three-step process that is performed from the dedicated web page (top screen). Once the tree file has been uploaded and the computations have been performed, the tree can be explored (bottom screen). This last screenshot shows the appearance of Lifemap for a fully bifurcating tree with nodes automatically named (see text).

In addition to the three precomputed trees described above and available online, Lifemap comes as a virtual machine that can be downloaded and used to visualize any tree. A web page (Fig 3) available from the browser once the virtual machine is running allows the user to upload a tree file (Step 1), perform all the necessary computations on the server (Step 2), and explore the tree (Step 3). On the exploration page, the user has access to a search bar that allows finding and zooming to any node or tip based on its name (Fig 3).

Because Lifemap requires nodes to be named, and because this condition is not met for most trees, the server version of Lifemap automatically gives names to unnamed nodes. This is done by traversing the whole tree from tips to root and assigning each (unnamed) node the names of two of the direct descendants, separated by a “+” sign.

## Discussion

Lifemap should become a useful source of information for the general public interested in evolution and biodiversity, but also for education and research in various fields related to ecology, evolutionary biology, and genomics. In addition, the Lifemap virtual machine might help researchers manipulating very large trees to visualize their data. Lifemap is available online at http://lifemap.univ-lyon1.fr. The general public version is also available as a mobile app for Android phones and tablets on the Play Store (as “Lifemap—Tree of Life”), and will soon be available for iOS devices and Windows phones.

## Materials and Methods

### Pipeline

A pipeline was written in Python (v2.7) to perform all the actions to create Lifemap and keep it up-to-date. The pipeline uses the ETE 3 toolkit [14] for tree operations (traversal) and NCBI interrogation. Here are the main steps:

- Retrieve NCBI taxonomy from the NCBI website (or OTOL taxonomy from the OTOL web site) and convert to Newick format.
- Only for the general public version: Remove all taxa whose name contains the words “unidentified,” “Unidentified,” “unclassified,” “Unclassified,” “environmental,” “Environmental,” “uncultured,” or “Uncultured,” and all taxa below the species level.
- While traversing the tree:
  - Compute the coordinates of (i) nodes and tips, (ii) half-circles, (iii) half-circle centers where clade names are written at some zoom levels, (iv) rank names along the half-circles
  - Write all these coordinates in a postgreSQL database that has PostGIS enabled
  - Write information associated with each node and tip into the Solr Search Engine v5.4.0 (scientific name, common name, coordinates, zoom level, rank name, taxid).
  - Get additional information for each taxa (for now, the number of genomes fully sequenced), and write this information into Solr.

All Lifemap functions are freely available (Under the GNU General Public License) on GitHub at https://github.com/damiendevienne/Lifemap/, along with detailed descriptions of the functions, the configuration files, the requirements, etc. Readers are referred to this source of information for more details.

### NCBI and OTOL data

The NCBI taxonomy data is retrieved from the NCBI ftp site (ftp://ftp.ncbi.nlm.nih.gov/pub/taxonomy/) every time the tree is updated. This is done automatically by the ETE 3 toolkit [14] used in the pipeline described above. For the Lifemap OTOL version, the latest OTOL synthetic tree is downloaded from https://tree.opentreeoflife.org/about/synthesis-release/, and the associated taxonomy version is retrieved from https://tree.opentreeoflife.org/about/taxonomy-version/. Currently, the synthetic tree version 7.0 is used, along with the taxonomy version 2.10.

### Tiling and image production

**Fig 4:**
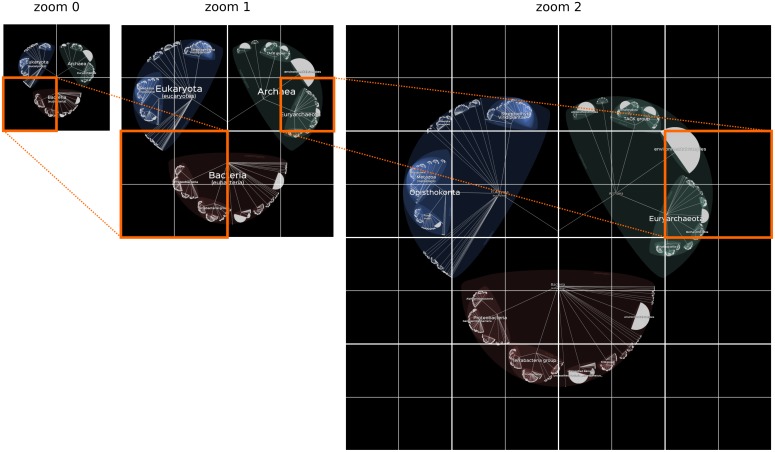
Principle of the tiling system used in Lifemap. Like in OSM, the image displayed at a given zoom level is composed of paving (or tiling) of small square images. To each of these square images at a given zoom level corresponds four images of the same size at the next level.

The zooming capability of the tool relies on the same principle and the same tools as OSM. The whole space is cut into square images (tiles) of 256 x 256 px and to each image at zoom *n* corresponds four images at zoom *n* + 1 (Fig 4). Images are generated when first requested by a user, thanks to the *mod_tile* open source apache module linked to the *renderd* tool (both part of the OSM suite of tools [15]) and cached on the server for further accession. The creation of the images is performed by *mapnik* v3.0.9 [16], which reads the database, formats the graphical elements with respect to a style sheet written for Lifemap, and returns the images to the server.

Note that the number of images is expected to grow exponentially. The level zero of Lifemap, where all three superkingdoms are visible, is formed by four images. Level one requires 16 images (4^2^), level two requires 64 images (4^3^), …, level *n* requires 4^*n*+1^ images (Fig 4). The number of zoom levels required to go to the deepest species in Lifemap-OTOL in its current version is 52, so if all images were generated at all zoom levels, the number of images would be around 1.1 x 10^32^ (Σ4^*i*^ for *i* in (1:53)), which is too big to be stored. However, the vast majority of these images are entirely black and are simply not generated, because images are generated on user's demand only, which solves the issue. The size of our servers is sufficient for all the images that are not entirely black to be generated and stored (estimated to be around 200Go for the different versions of Lifemap).

### Map–browser interaction and mobile version

The websites on which Lifemap can be explored were written in HTML5, CSS, and Javascript, using the Leaflet v0.7 javascript library [17]. A mobile version of the tool was also written. The user is automatically redirected to this website if accessing Lifemap from a phone.

The creation of the mobile app was performed using PhoneGap [18], a tool that allows encapsulating an HTML5/CSS/Javascript website and all its dependencies into a fully featured mobile application that can be uploaded to Android, Apple, or Microsoft stores.

## Abbreviations

MRCA: most recent common ancestor
OSM: OpenStreetMaps
OTOL: Open Tree of Life
ToL: Tree of Life
